# Epigenetic legacy of early-life undernutrition: methodological lessons from Dutch and Chinese famine studies

**DOI:** 10.3389/fgene.2026.1804465

**Published:** 2026-04-10

**Authors:** Hasan Khatib, Elijah Ellsworth, Norisha Badhwar

**Affiliations:** Department of Animal and Dairy Sciences, The University of Wisconsin-Madison, Madison, WI, United States

**Keywords:** Chinese famine, Dutch famine, epigenetic methodology, research design, transgenerational inheritance

## Abstract

The recent rise in global food insecurity has renewed scientific interest in understanding the long-term health consequences of early-life nutritional deprivation. This study critically evaluates the experimental designs and methodological approaches of key publications examining the epigenetic and phenotypic effects of the Dutch and Chinese famines. Specifically, these studies were assessed for sample size, control group selection, relevance of tissue sampling, timing of famine exposure, and the quality of statistical reporting. Research on both famines has centered on prenatal exposure and subsequent health outcomes, providing important insights into how *in utero* nutritional deprivation may lead to long-lasting epigenetic modifications. These changes have been linked to elevated risks for metabolic, cardiovascular, and neuropsychiatric disorders. Despite these contributions, many studies exhibited notable limitations, including small sample sizes, questionable accuracy in reporting health outcomes, and issues with the selection of control groups. Such methodological shortcomings may have led to the misinterpretation of some findings. Ongoing and recent famines in regions such as Sudan, Somalia, and Gaza—driven by conflict and environmental disasters, including droughts and floods—represent some of the most pressing humanitarian crises of our time. Lessons from studies of the 20th-century Dutch and Chinese famines can inform the design of future research on the biological and intergenerational consequences of famine and trauma. Improved study designs will enhance the ability to generate reliable evidence and guide global health strategies for populations at risk of transgenerational effects from nutritional deprivation.

## Introduction

Environmental exposures, including nutritional constraints, stress, chemicals, and toxicants, are known to modify the epigenome, consequently influencing disease susceptibility and other phenotypic outcomes across subsequent generations in humans and experimental animals (Jirtle and Skinner, 2007). Several groundbreaking studies have demonstrated that environmental factors, including maternal diet and exposure to endocrine disruptors during pregnancy, can induce phenotypic and epigenetic changes in offspring (Anway et al., 2005; Blewitt et al., 2006; Dolinoy et al., 2006; Morgan et al., 1999). Environmental epigenetics studies indicate that the effects of parents' experiences, including undernutrition or stress, can be passed to their offspring through intergenerational or transgenerational epigenetic inheritance mechanisms.

According to the United Nations World Food Program, the world is facing an ongoing global food crisis driven by four main factors: conflict, climate change, economic instability, and displacement (https://www.wfp.org). Among these, conflict is the leading cause, as it destroys infrastructure, displaces populations, fuels inflation, and restricts access to markets, ultimately resulting in widespread hunger and food insecurity. In turn, hunger can fuel social unrest, as communities protest about rising food prices or compete for limited resources.

At the same time, extreme weather events are becoming more frequent and severe, disrupting agriculture and significantly affecting both local and global food systems. In 2021, climate-related disasters were the leading cause of severe hunger in eight African countries, pushing 23.5 million people into critical levels of food insecurity (https://www.wfpusa.org).

According to the 2023 Global Report on Food Crises (GRFC), nearly 282 million people across 59 countries and territories faced severe acute hunger, representing an increase of 24 million from the previous year. This rise is attributed to both expanded reporting and a worsening food security situation, particularly in regions such as the Gaza Strip and Sudan (https://www.wfpusa.org). Global food demand is projected to rise by more than 50% by 2050; however, climate change is expected to reduce agricultural yields of major crops over the same period (https://www.csis.org). This imbalance could lead to sharp increases in food prices, heightened food insecurity, social unrest, and political instability—conditions that may escalate into conflict and trigger famine-like scenarios (https://2021-2025.state.gov). To better anticipate the potential consequences of such crises, it is crucial to study historical famines that highlight the impacts of nutritional deprivation. Two of the most frequently examined cases are the Dutch Famine of 1944 and the Chinese Famine of 1959. Importantly, these famines were chosen for this study because they represent the only human famine cases with available epigenetic data.

The Dutch Famine, commonly known as the 'Hunger Winter' (Hongerwinter), occurred between November 1944 and April 1945 during the final stages of World War II. It severely impacted the western region of the Netherlands, particularly major cities such as Amsterdam, Rotterdam, and Hague (Lumey and Van Poppel, 1994). The immediate cause of the famine was a German blockade, imposed in retaliation for a railway strike organized by the Dutch resistance in support of the advancing Allied forces. The blockade cut off food and fuel supplies to the affected regions, further straining an already fragile wartime economy (Lumey et al., 2007). As a result, the population experienced severe malnutrition, with daily caloric intake dropping to as low as 400–800 calories (Mihai et al., 2015). An estimated 20,000 people died, and approximately 4.5 million were affected by the direct and indirect consequences of the famine (Conti et al., 2024). The famine had lasting demographic and health effects, particularly among individuals exposed *in utero* or during adolescence. The famine officially ended in May 1945 with the liberation of the Netherlands by Allied forces (Stein and Susser, 1975). However, its legacy persists, as subsequent generations continue to experience consequences linked to parental exposure, effects associated with epigenetic modifications.

The Dutch famine presented a unique opportunity to examine David Barker’s fetal origins hypothesis and the multigenerational effects of hunger on subsequent generations. Prenatal exposure to famine during early gestation correlated with hypertension, elevated blood pressure, and a notable rise in coronary heart disease later in adulthood, thereby substantiating the Barker hypothesis (Roseboom et al., 2011). Moreover, women who experienced famine during pregnancy gave birth to offspring with increased neonatal adiposity and compromised health, indicating the potential transgenerational transmission of maternal undernutrition effects (Roseboom et al., 2011). To investigate whether prenatal exposure to the Dutch famine resulted in epigenetic alterations, DNA methylation levels of insulin-like growth factor II (*IGF2*) were evaluated in individuals conceived during the famine 6 decades earlier, with unexposed same-sex siblings serving as controls (Heijmans et al., 2008). The exposed individuals showed lower methylation levels. The Dutch famine studies show that maternal undernutrition during both the early and late stages of pregnancy can affect offspring health.

The Great Chinese Famine has been largely attributed to policies implemented by the Chinese Government during the “Great Leap Forward” in the mid-20th century. This campaign introduced unproven agricultural techniques and prioritized rapid industrialization, diverting labor from farming and severely disrupting food production. As a result, grain output declined sharply—by more than 25% at its peak, equivalent to a reduction of 53 million tons—triggering widespread famine across China (Wang et al., 2019). The death toll is estimated to have exceeded 30 million people by 1961 (Smil, 1999), and the famine caused a dramatic decline of more than 20% in the birth rate over the following 5 years (Jowett, 1991). Beyond the death toll, the famine had profound effects on physical and mental health, cognition, and other severe consequences for both those who experienced it and their offspring. Exposure to famine between the ages of three and five was linked to mental health issues, resulting in approximately eight million additional cases of depression in adulthood (Cheng et al., 2024). Furthermore, fathers who were affected by malnutrition during the famine had children with impaired cognitive abilities (Tan et al., 2023). Interestingly, exposure to poor nutrition *in utero* and during early adulthood during the Chinese famine was associated with reduced height in two successive generations (Yao et al., 2024).

Research on the epigenetic effects of the Dutch and Chinese famines has primarily focused on increased health risks associated with prenatal exposure to famine. While numerous studies have explored these outcomes, our objective is to critically evaluate the experimental designs underpinning these studies. Specifically, we assessed whether Chinese and Dutch famine studies employed appropriate controls, had sufficient sample sizes, defined clear famine exposure windows, and collected accurate phenotypic measurements, all of which are essential for ensuring the validity and interpretability of their conclusions. This analysis is significant in identifying the methodological strengths and limitations of existing famine research, thereby guiding future studies toward more rigorous and informative designs. Ultimately, improved methodologies will enhance our understanding of how prenatal famine exposure affects long-term disease risk, with significant implications for public health planning and policy development in the context of future nutritional crises.

## Methods

We conducted a comprehensive literature search to identify studies examining the epigenetic consequences of early-life undernutrition, with a particular focus on famine-exposed cohorts. Searches were performed primarily in PubMed, supplemented by Google Scholar to ensure broad coverage of relevant biomedical, epidemiological, and methodological literature. Search terms included combinations of keywords such as famine, early-life undernutrition, prenatal exposure, epigenetics, DNA methylation, developmental origins of health and disease, Dutch Hunger Winter, and Chinese famine. Reference lists of key articles and relevant reviews were manually screened to identify additional studies. Citation tracking was used to capture influential and critical methodological papers.

This review does not aim to provide a meta-analysis of all studies on the Dutch and Chinese famines. Instead, 21 representative papers were selected for their focus on the effects of famine exposure on both the exposed individuals and subsequent generations, with particular emphasis on studies exploring epigenetic mechanisms, especially DNA methylation. However, due to the limited number of available studies, all relevant papers addressing intergenerational effects of famine were included, regardless of their specific epigenetic focus. This inclusive and systematic approach ensured a robust foundation for evaluating the long-term biological consequences of famine exposure.

## Results and discussion

The analysis of the Dutch and Chinese famine studies involved several critical considerations, including epigenetic analysis to detect associations between famine outcomes and epigenetic marks, tissue selection for epigenetic and metabolic analyses, sample size, and the timing of famine exposure. Table 1 provides an overview of epigenetic and metabolic outcomes associated with prenatal exposure to the Chinese and Dutch famines.

**TABLE 1:** Key epigenetic and metabolic findings from studies of prenatal exposure to the Chinese and Dutch famines.

References	Study design	Key findings	Limitations
Zeng et al. (2023)	Sample size: 184 subjects Controls: 105 subjects born post-famine (January 1, 1963 - 1 Dec 1964) Tissues: Whole blood	The study examined prenatal famine effects on DNA methylation in serotonin signaling genes but was limited by the lack of an appropriate control group Significant hypomethylation of serotonin receptor signaling pathway genes was observed in females (p = 0.019), but not in males Gene-specific hypomethylation was detected in *HTR2A* (p = 0.033) and *HTR2C* (p = 0.014) in females only The findings provide early human evidence linking prenatal famine to sex-specific epigenetic changes in psychiatric-related genes	The “control” group includes only post-famine individuals, with no pre-famine comparison, limiting the ability to isolate famine-specific effects DNA methylation was measured in whole blood rather than brain tissue, which is more relevant for serotonin receptor pathway effects
He et al. (2019)	Sample size: 79 subjects Control: “Unexposed” but did not provide dates Tissue: Whole blood *In vitro* validation: 10 fibroblast cultures from 5 donors subjected to 72-h nutritional deprivation	The study identified three significant differentially methylated regions (DMRs) in promoter regions of *ENO2*, *ZNF226*, *CCDC51*, and *TMA7* (P < 0.005) These genes are involved in nervous system development and neurogenesis, suggesting that famine exposure may influence developmental processes through epigenetic modification of gene promoters	The sample size is small (79 total; 25 exposed), limiting power to detect genome-wide significant differences after multiple testing correction The fibroblast findings may not translate to human famine survivors. Also, differences in blood cell composition may affect the results
Li et al. (2021)	Sample size: 186 Control group: 105 born post-famine (January 1, 1963 - 31 December 1964) Tissue type: Whole blood	The study examined famine effects on DNA methylation in metabolic genes, identifying significant hypomethylation of *EHMT1*, *CNR1*, *UBXN7*, and *ESM1* associated with fetal exposure These genes are linked to neurodevelopment, neurological disorders, and vascular function, suggesting famine-related epigenetic effects on neurological and vascular health	A ∼4-year age difference between exposed and unexposed groups led to the removal of many age-associated CpGs, potentially masking relevant sites Whole blood is not ideal for assessing methylation in brain tissue. tissue.
Shen et al. (2019)	Sample size: 188 subjects Exposed: Subjects born during famine (1959–1961) in Anhui province (severe famine) Control: Combined subjects born pre-famine (1956–1958) and post-famine (1962–1964) Tissue type: Whole blood	Early-life famine exposure was associated with increased *IGF2* CpG1 methylation (β = 0.07, P = 0.0008) and higher total cholesterol (β = 0.72, P = 1.09 × 10^−7^) Each unit increase in CpG1 methylation was linked to a 1.09-unit increase in cholesterol (P = 0.03) CpG1 methylation explained 5% of the famine–cholesterol association, suggesting a modest epigenetic contribution to long-term metabolic effects	The mediation effect of IGF2 CpG1 accounted for only 5% of the famine–cholesterol association, leaving most of the effect unexplained
Wang et al. (2010)	Sample size: 17,023 Toddler exposed (1956–1958) n = 4,563 Gestational exposed (959–1961) n = 4,056 Controls (1962–1964) n = 8,404 Tissue type: N/A (anthropometric measurements only)	Famine exposure was linked to shorter, overweight females and shorter, slimmer males, indicating sex-specific long-term effects	Samples were drawn only from employed individuals attending routine health checkups, introducing selection bias. Those most severely affected by obesity may have been unable to maintain employment and were therefore excluded
Boks et al. (2018)	Sample size: 153 subjects (Chinese famine cohort) Exposed: 48 subjects (23 schizophrenia patients, 25 controls) exposed to famine in the first trimester (born January 1960-September 1961) Controls: 105 unexposed subjects (51 schizophrenia patients, 54 controls) Tissues: Whole blood, postmortem brain (N = 214), fibroblasts (N = 10) Additional validation samples: Dutch case-control blood (N = 64), postmortem brain tissue (N = 214)	The study found significant *DUSP22* promoter hypermethylation in famine-exposed patients with schizophrenia and in patients with schizophrenia regardless of famine exposure Hypermethylation was replicated in unexposed Dutch patients in blood and postmortem brain Chromosome 16 genetic regulation and fibroblast experiments confirmed increased *DUSP22* methylation and expression under nutritional deprivation, suggesting genetic vulnerability mediates famine–schizophrenia links	Sample sizes are small across all cohorts, particularly in the case–control blood samples, limiting statistical power The lack of correlation between DUSP22 methylation and gene expression in brain tissue limits the functional interpretation of the findings Fibroblast models may not fully capture the *in vivo* complexity of famine exposure Medication use and smoking may confound the results
Wang et al. (2019)	Sample size = 235 subjects Control group: Combined pre-famine exposed (January 1, 1958 - 1 Dec 1958) and post-famine (October 1, 1962 - September 30, 1964). Total = 160 non-exposed individuals Famine-exposed group = 75 individuals born during the famine Tissues: Whole blood	Prenatal famine exposure was linked to increased INSR methylation—particularly at CpG7—and larger adult waist circumference, but not BMI. CpG7 partially explained the association between famine and waist No changes were observed in IGF2, suggesting that INSR epigenetic modifications may underlie the long-term risk of central obesity	DNA methylation was measured in whole blood, which may not reflect relevant tissues such as the liver or adipose tissue Methylation was assessed 5 decades after exposure, potentially obscuring the original famine-induced effects
Yao et al. (2024)	Sample size: 5401 F1 participants (born 1955–1966) and 3930 F2 offspring Exposed: 3222 F1 subjects exposed to Chinese famine (1451 fetal-exposed, 1771 childhood-exposed) Controls: 2179 unexposed F1 subjects (born 1963–1966) Tissues: Height measurements in adulthood for F1 and F2; repeated height measurements during childhood for F2 (<18 years) Additional validation samples: Paired parents (1660 couples) with 1171 adult offspring and 2512 child offspring	F1 individuals exposed to famine *in utero* or childhood were shorter in adulthood (fetal: −0.41 cm; childhood: −1.12 cm) F2 offspring of childhood-exposed fathers or both parents exposed showed reduced height in childhood and adulthood, with stronger effects for paternal childhood exposure and highly educated F1 parents, indicating intergenerational transmission of famine effects	Famine exposure was assigned based on birth year rather than on individual nutritional status F2 offspring of unexposed parents were born later than those of exposed parents, potentially inflating height differences
Gørgens et al. (2012)	Sample size: Not explicitly stated, but includes multiple cohorts from 1938 to 1971 Controls: Individuals born in pre-famine (1938–1947) and post-famine (1962–1971) years Tissue type: Not applicable (study focused on height and socioeconomic data)	Early-life famine caused significant stunting among rural individuals, especially among younger individuals Positive selection among survivors partially offset this, as their children were taller than expected, making adult height a complex indicator of economic wellbeing	The sample size is not reported, making it difficult to assess statistical power Famine exposure was assigned based on birth region rather than on individual nutritional status, which may obscure true effects The model does not account for epigenetic inheritance, focusing only on the direct effects of famine on height
Roseboom et al. (2011)	Sample size: 2,060 births Controls: Babies born before 7 January 1945 Tissue: Placenta	Amine disrupted placental development, reducing area and altering efficiency Effects varied by gestational timing and sex: late-gestation exposure thickened placentas in girls but reduced overall efficiency, whereas early-gestation or post-famine conception improved efficiency and increased birth size, highlighting sex-specific adaptations	The control group included babies born before 7 January 1995, who were not directly exposed to famine but may still carry famine-related epigenetic effects Placental weight—a standard measure of placental size and efficiency—was not assessed Reliance on area and derived estimates provides only indirect measures and may not capture functional differences Potential selection bias and wartime-specific confounding factors may also affect the results
Ravelli et al. (1998)	Sample size: 741 individuals born a year before and after the famine Used weight measurements for mothers	Early-gestation famine exposure was linked to higher weight, BMI, and waist circumference at age 50 in women, but not men, independent of birth size Poor early-pregnancy nutrition followed by recovery may drive long-term obesity in women, highlighting sex- and timing-specific effects of prenatal undernutrition	Controls conceived after the famine may have been indirectly exposed through maternal oocyte exposure Famine exposure was estimated from official rations, which may not reflect actual maternal intake
De Groot et al. (2011)	Sample size: 2,417 births between 1 February 1945, and 31 March 1946 (infants whose mothers were 2 months pregnant when famine started, or who conceived during famine, or in the months following its end) Time controls: 890 births from 1943 to 1947 (infants whose mothers did not experience famine) 324 same-sex siblings in individuals who responded	Gestational famine exposure had no significant impact on cognitive function Early gestation exposure showed a modest, non-significant decline in scores, while later exposure had a slight positive effect on one measure Results were consistent across models, with no sex-specific effects	Exposure was defined by official rations rather than individual maternal intake Controls conceived after the famine may have had oocyte exposure While the overall sample is large, dividing by gestational windows reduces group sizes, especially weeks 1–10 (n = 74), limiting power to detect subtle cognitive changes
Heijmans et al. (2008)	Sample size: 60 individuals conceived during the famine (periconceptional exposure) and 62 individuals exposed in late gestation Controls: Same-sex siblings of exposed individuals served as controls Tissue: Whole blood	Periconceptional famine exposure during the Dutch hunger winter was associated with significantly lower *IGF2* DMR methylation in adulthood (−5.2%), confirmed in a subset. Later-gestation exposure did not affect methylation but was linked to lower birth weights These findings highlight the critical role of exposure timing, with periconceptional famine having a stronger impact on *IGF2* methylation than age-related changes	Controls conceived after the famine may have had oocyte exposure The small sample size limits power, generalizability, and the ability to detect subtle effects Methylation was measured in blood, which may not reflect tissue-specific changes in relevant organs Postnatal environmental differences among sibling controls could also affect results
Franzek et al. (2008)	The study population consisted of native Dutch addicted patients registered in the Rotterdam addiction Treatment program and controls, who were native Dutch inhabitants of Rotterdam, all born between 1944 and 1947 Controls: individuals conceived in the equivalent period in the subsequent year	First-trimester famine exposure was linked to higher addiction risk in later life, especially in men, highlighting this period as a critical window for neurodevelopmental influences	Individuals born after the famine may not have been fully unexposed due to preconception or parental effects Overlap between controls and affected individuals could bias associations Cases came from an addiction treatment program, limiting generalizability to all affected individuals Lack of comorbidity data makes it difficult to separate addiction from other mental health disorders
Hughes et al. (2009)	Cohort: 58,279 men and 62,573 women between the ages of 55 and 69 Used a self-administered questionnaire Sub-cohort: 5,000 individuals randomly selected from the cohort Tissue: Tumor-specific tissue	Adolescent energy restriction during the 1944–45 hunger winter was linked to a lower risk of CIMP-type colorectal cancer, consistent across rural and urban areas, but showed no effect on MSI-type tumors	No true unexposed control group Energy restriction was estimated from father’s employment, residence, and historical period, which may not reflect actual intake, introducing possible misclassification bias Father’s employment also reflects socioeconomic status, complicating the isolation of famine effects Methylation was measured in tumor rather than normal tissue, limiting causal interpretation
Tobi et al. (2014)	Sample size: 48 individuals (24 same-sex sibling pairs) Control group: Unexposed same-sex siblings, involved babies born before or conceived after the famine Tissue: Whole blood	Periconceptional famine exposure was linked to 181 differentially methylated regions enriched in regulatory elements, with six validated genes (*SMAD7, CDH23, INSR, CPT1A, KLF13, RFTN1*) *INSR* was associated with birth weight, and CPT1A with LDL cholesterol, suggesting that early gestational exposure may cause modest methylation changes that affect long-term metabolic outcomes	Sibling controls born after the famine may not have been fully unexposed due to preconception or parental effects RRBS measures only a subset of CpG sites, with a bias toward CG-rich regions Methylation was assessed in whole blood rather than in metabolically relevant tissues such as the liver, pancreas, or adipose tissue
Tobi et al. (2009)	Sample size: 244 subjects: Selected from births between 1943 and 1947 −60 periconceptually exposed individuals +60 same-sex sibling controls −62 late-gestation exposed individuals +62 same-sex sibling controls Controls: 311 births and 311 unexposed siblings Tissue: Whole blood	Prenatal famine exposure caused persistent, timing- and sex-specific DNA methylation changes at growth- and metabolism-related genes Periconceptional exposure decreased *INSIGF* methylation and increased *IL10, LEP, ABCA1, GNASAS,* and *MEG3*, with sex-specific effects for *INSIGF, LEP,* and *GNASAS*. Late-gestation exposure altered *GNASAS* and *LEP* methylation in males, indicating lasting epigenetic effects on disease risk	Fifteen preselected loci were analyzed, limiting detection of genome-wide methylation changes Methylation was measured in whole blood rather than in relevant tissues
Stein et al. (2006)	Sample size: 375 same-sex siblings of both exposed and unexposed individuals Controls: Individuals born before or conceived after the famine	Gestational famine exposure was associated with higher blood pressure and a 44% increased risk of hypertension at age 59 Exposure ≥10 weeks raised systolic by 2.77 mmHg and diastolic by 1.27 mmHg Higher birth weight lowered blood pressure, but overall gestational undernutrition predisposed offspring to later hypertension	Controls conceived after the famine may not have been fully unexposed due to preconception or parental effects
Roseboom et al. (2011)	Sample size: 2414 singleton live births (741: Clinical examination, 912: Health interviews) Controls: Individuals born before or conceived after the famine Clinical health parameters assessed	Chronic diseases may originate from fetal adaptations to undernutrition, with long-term effects depending on exposure timing and tissue development Coronary risk factors—impaired glucose tolerance, high cholesterol, hypertension, and obesity—can be programmed *in utero*, and maternal malnutrition can impact adult health even without affecting birth size	Controls conceived after the famine may not have been fully unexposed due to preconception or parental effects
Hulshoff Pol et al. (2000)	Sample size: 36 (9 exposed schizophrenia patients, 9 healthy exposed controls, 9 unexposed schizophrenia patients, and 9 unexposed controls) Controls: Individuals born before or conceived after the famine (1941–1944 or 1946–1949) Brain imaging via MRI	First-trimester famine exposure was linked to increased brain abnormalities, especially white matter hyperintensities, and reduced intracranial volume in individuals with schizophrenia Early gestational undernutrition may drive structural brain changes and elevate schizophrenia risk, potentially interacting with genetic susceptibility	Small sample size (n = 36; 9 per subgroup), limiting power, and generalizability Controls conceived after the famine may not have been fully unexposed due to preconception or parental effects
Wiegersma et al. (2022)	Sample size: 595 participants Controls: Individuals born before or conceived after the famine (born before 7 January 1945, or conceived after 8 December 1945) Used self-reported cognitive assessments and questionnaires	Prenatal famine exposure was linked to increased cognitive problems at age 72, with timing- and sex-specific effects: Late-gestation exposure affected women, early-gestation exposure affected men Early fetal undernutrition may have lasting impacts on brain function and late-life cognitive health	The study relied on self-reported outcomes rather than objective neurophysiological testing or clinical diagnoses Results may be influenced by reporting bias and confounding factors such as anxiety or depression Controls conceived after the famine may not have been fully unexposed due to preconception or parental effects

### Famine exposure assessment

A crucial consideration in famine studies, particularly regarding prenatal exposure, is the timing of exposure during specific gestational periods. Several Dutch studies define famine exposure by early, mid, or late pregnancy. For instance (Roseboom et al., 2000), in their analysis of lipid profiles, they categorized individuals into three gestational exposure groups: early (born August 19–8 December 1945), mid (born April 29–18 August 1945), and late gestation (born January 7–28 April 1945). Similarly, Ravelli et al. (Ravelli et al., 1998), focusing on glucose metabolism, used the same three 16-week intervals to classify late (January 7–April 28), mid (April 29–August 18), and early gestation (August 19–December 8) exposure. Research on cognitive function has also followed this gestational categorization (Wiegersma et al., 2022), reinforcing the importance of developmental timing in assessing the long-term effects of prenatal famine exposure.

Most Chinese famine studies utilized a cohort-based design to compare individuals born during the famine period (1959–1961) with those born post-famine (1962–1964). Although these comparisons may provide useful information for contrasting famine and post-famine cohorts, some control groups (Li et al., 2021; Zeng et al., 2023) included individuals born soon after famine recovery, thereby increasing the risk of residual maternal nutritional stress and reducing the ability to detect biologically meaningful differences between groups. Control groups, such as those used in (Wang et al., 2019; Shen et al., 2019) included pre-famine cohorts (1956–1958), which may be more informative as a baseline for comparison of permanent environmental and epigenetic effects.

In the Dutch Famine studies, some control groups included individuals conceived after the famine, an approach that may introduce confounding factors related to post-famine recovery and environmental changes (De Groot et al., 2011; Franzek et al., 2008; Heijmans et al., 2008; Ravelli et al., 1998; Roseboom et al., 2000; Tobi et al., 2014). However, the use of controls conceived after famine introduces the possibility that observed epigenetic changes may reflect modifications present in the oocyte before fertilization. This raises the potential for confounding due to maternal germline exposure, complicating the interpretation of results attributed solely to famine effects on the fetus.

In contrast, some studies lacked a well-defined measure of famine exposure, potentially biasing their findings. For example, the study by Hughes et al. (2009) examined the impact of famine exposure on colorectal cancer risk in the Dutch population. They found that energy restriction during adolescence and early adulthood was associated with a reduced risk of developing tumors marked by promoter methylation in five specific genes. However, the criteria used to define famine exposure—such as the father’s place of residence during the famine, the individual’s residence during World War II, and employment status—could have introduced bias. As the authors acknowledged, these proxy indicators may have led to misclassification of exposure. In a separate study, Franzek et al. (2008) examined the association between prenatal famine exposure and addiction later in life among individuals in Rotterdam, the Netherlands. They reported that exposure to famine during the first trimester was associated with an increased risk of addiction in adulthood. However, a significant limitation of the study was the uncertainty regarding whether the patients and controls were actually born in the Rotterdam area, making it unclear if they had been exposed to the famine. Additionally, the authors acknowledged the lack of information on the health status of the control group, which further limited their ability to rule out potential bias in the results.

In summary, inconsistencies in how famine timing and severity are defined across studies call into question the robustness of many conclusions (Li et al., 2019). For example, the Chinese famine varied substantially in onset and duration across provinces, and its severity differed markedly between rural and urban regions. These discrepancies led Li and colleagues (Li et al., 2019) to argue that conclusions linking prenatal famine exposure to type 2 diabetes in Chinese cohorts require careful re-evaluation.

### Tissue selection

Selecting the appropriate tissue for analysis is crucial in famine research, as it enables an accurate assessment of intergenerational changes and more precise predictions of genotype-to-phenotype relationships, given the variability in gene expression patterns and functional roles across different tissues. Tissue selection varied across studies, affecting the interpretation of famine effects on epigenetic modifications.

Blood samples were the primary medium for DNA methylation analysis (He et al., 2019; Shen et al., 2019; Wang et al., 2019; Zeng et al., 2023). Indeed, most authors acknowledge that using peripheral blood cells decades after famine may not reflect the actual DNA methylation patterns in the relevant tissues. For example, it would be challenging to interpret the association between DNA methylation profiles and brain disorders using blood DNA methylation in the absence of knowledge of the shared gene expression between blood and brain. He et al. (2019) simulated famine conditions using fibroblast cell cultures derived from skin biopsies obtained from healthy Dutch individuals. They analyzed these cell lines for DNA methylation and compared the results with those obtained from DNA methylation profiles of Chinese individuals with a famine history. Three DMRs located in genes involved in nervous system development were common between fibroblasts and blood cells. A major limitation of this study is the selection of fibroblasts and blood cells for DNA methylation analysis, as these tissues, as the authors acknowledge, may not reflect brain DNA methylation patterns. In addition, the comparison of DNA methylation patterns obtained from fibroblast cell culture and blood samples collected 50 years after famine exposure raises questions about the validity of the results. In contrast, Boks et al. (2018) incorporated both blood and postmortem prefrontal cortex samples to investigate the effects of famine on the DNA methylation levels of the DUSP22 gene in individuals with schizophrenia and controls. They reported higher methylation levels of this gene in patients with schizophrenia exposed to famine compared with healthy famine-exposed controls and healthy non-exposed controls. The authors also used fibroblast cell lines nutritionally deprived to mimic famine conditions.

Blood samples have also been used in metabolic and cardiovascular research (Heijmans et al., 2008; Tobi et al., 2014), offering insight into systemic changes in gene regulation. However, blood may not fully capture tissue-specific epigenetic effects, as its heterogeneous cell composition (e.g., lymphocytes, neutrophils) can obscure or confound methylation signals. Moreover, DNA methylation changes detected in blood cells may not accurately reflect the epigenetic consequences of prenatal famine exposure in relevant target tissues. The reliance on blood or fibroblast cell lines may, therefore, limit the interpretability and biological relevance of these studies. Epigenetic regulation is highly cell- and tissue-specific, reflecting differences in developmental lineage and gene regulatory programs. Comparative analyses have revealed substantial variation in DNA methylation patterns across tissues, indicating that epigenetic marks detected in blood may not accurately reflect regulatory states in organs such as the brain, liver, pancreas, or adipose tissue (Roadmap et al., 2015). Nevertheless, due to practical constraints in sampling internal organs in humans, blood-based methylation data have been widely used to study diseases unrelated to blood cells, including neurodevelopmental disorders (Zhan et al., 2026) and metabolic conditions such as type 2 diabetes and obesity (Chambers et al., 2015). However, interpreting these studies remains challenging. Cross-tissue comparisons show that correlations between blood methylation and disease-relevant tissues are often modest and highly locus-specific, with many CpG sites exhibiting limited concordance between blood and tissues such as the brain (Hannon et al., 2015). As a result, methylation differences detected in blood may reflect systemic responses, exposure biomarkers, or shifts in immune cell composition rather than causal regulatory mechanisms in the tissues directly driving disease.

An additional limitation arises from the cellular heterogeneity of peripheral blood, which comprises multiple immune cell types with distinct methylation profiles. Variation in cell-type composition across individuals or disease states can therefore produce apparent methylation differences unrelated to regulatory changes within specific cell populations. Although statistical methods have been developed to adjust for cell mixture effects (Houseman et al., 2012), residual confounding may persist. Together, these considerations underscore the need to interpret blood-based epigenetic associations cautiously and, where possible, to integrate analyses from biologically relevant tissues to better elucidate the mechanisms linking environmental exposures, such as famine, to long-term phenotypic outcomes.

### Selection of controls and statistical power

The selection of case and control groups is critical for understanding the effects of prenatal famine exposure. Most studies employed a cohort-based design, comparing individuals exposed to famine *in utero* with those conceived before or after the famine. Interestingly, one particular study examining prenatal exposure for colorectal cancer risk did not utilize a control group (Hughes et al., 2009), weakening the validity of the study and undermining its ability to draw solid conclusions. Another relevant study is that by Franzek et al. (2008), which investigated the relationship between prenatal famine exposure and later-life addiction. The study may have misclassified some individuals with addiction as controls, potentially underestimating the true association. Additionally, the authors acknowledged their inability to verify whether all participants, both cases and controls, were born in the Rotterdam area during the famine. This uncertainty raises concerns about exposure misclassification, particularly among individuals born outside the famine-affected zone, potentially further biasing the results. In contrast, some studies employed same-sex sibling controls who were conceived after exposure of maternal oocytes (Gørgens et al., 2012; Heijmans et al., 2008; Stein et al., 2006; Tobi et al., 2014). This approach enhanced the reliability of findings by reducing genetic and environmental variability. However, residual maternal stress remained a potential confounding factor and limitation.

Several studies used post-famine cohorts as controls to assess the long-term phenotypic and epigenetic effects of famine exposure (Shen et al., 2019; Wang et al., 2019; Zeng et al., 2023). However, these controls were typically 3–5 years younger than the famine-exposed individuals, introducing substantial age-related bias (Li et al., 2019). Notably, re-evaluation of the association between famine exposure and type 2 diabetes using age-biased controls showed no increase in diabetes risk (Li et al., 2019).

Difficulties in recruiting participants for famine-related research have led to considerable variation in sample sizes across studies, which affects the statistical robustness of the findings. Large-scale epidemiological research, such as that by (Wang et al., 2010) (n = 17,023), investigated the effects of the famine on individuals born before the famine, those born during the famine, and those born after the famine. They identified strong population-level associations between prenatal famine exposure and an increased risk of short stature and overweight. These findings suggest that exposure to famine during early development has long-term consequences for metabolic health. In contrast He et al. (2019), acknowledged that the small sample size used in the DNA methylation analysis (25 famine-exposed individuals versus 54 unexposed individuals) lacked the statistical power to identify genetic markers associated with famine exposure. However, other factors could limit power, such as the use of blood samples collected decades after the famine and the lack of additional phenotypes collected for the examined population beyond famine exposure status. Similarly Shen et al. (2019), also acknowledged that the small sample size used in their analysis could have limited the generalizability of their results.

Another important consideration across famine studies is the variation in sample sizes, which influences both statistical power and the generalizability of findings. For example Heijmans et al. (2008), examined DNA methylation in the IGF2 gene in 60 individuals prenatally exposed to famine and 60 same-sex sibling controls. In contrast Hulshoff Pol et al. (2000) investigated the association between prenatal famine exposure and schizophrenia using a smaller cohort of 36 patients and matched controls. On the other hand, De Groot et al. (2011) conducted a larger study assessing cognitive function in 951 exposed individuals compared with 890 controls at age 59. Larger sample sizes, such as those in Roseboom et al. (2000) (n = 704), provided greater statistical power to detect associations, including a significantly higher LDL: HDL ratio in individuals exposed during early gestation. Similarly De Groot et al. (2011) reported significantly lower General Cognitive Index (CGI) scores in individuals exposed during early gestation, suggesting potential long-term cognitive impacts of famine-related exposure.

In summary, careful construction of control groups is essential for producing valid and interpretable findings in famine studies. Future research should always include well-defined control groups with verified absence of exposure and outcomes, ensuring accurate classification of both exposure status and disease phenotypes. Controls should be closely matched in age to avoid bias, and, whenever possible, family-based designs, such as same-sex sibling comparisons, should be used to reduce genetic and shared environmental variability, while still accounting for residual confounding factors. Incorporating multiple comparison groups (e.g., pre-, during-, and post-famine cohorts) can further strengthen causal inference. In addition, adequately large and representative samples are critical to ensure sufficient statistical power and generalizability, and matching or adjusting for key demographic and environmental factors is necessary to minimize confounding. Overall, rigorous control selection, careful matching, and sufficient sample size are fundamental to improving the reliability and interpretability of future famine research.

### Epigenetic analysis

Wang et al. (2019) investigated the impact of the Chinese famine exposure on the insulin receptor (INSR) gene by comparing three distinct birth cohorts defined by their timing of famine exposure. The pre-famine-exposure group consisted of individuals born in 1958 (n = 82), while the post-famine-exposure group included those born between 1 October 1962, and 30 September 1964 (n = 78). The main famine-exposed group comprised individuals born between 1 October 1959, and 30 September 1961, during the famine’s height (n = 75). Epigenetic analysis of blood samples focused on DNA methylation levels in the INSR gene. Phenotypic measures included body mass index (BMI), waist circumference, fasting plasma glucose levels, triglycerides, low-density lipoprotein cholesterol (LDL-C), high-density lipoprotein cholesterol (HDL-C), and diastolic and systolic blood pressure. This study found a link between early-life nutritional deprivation and subsequent epigenetic changes that are associated with metabolic health in adulthood. Individuals exposed to the Chinese famine during the prenatal period had higher waist circumference than non-exposed individuals (P = 0.029). Specifically, the fetal-exposure group showed significantly higher DNA methylation levels (+3.3%) in INSR (P = 0.003). Additionally, INSR DNA methylation was associated with increased triglyceride concentrations and decreased HDL-C levels. However, this study and similar undernutrition studies have several limitations, some of which the authors acknowledge. A major limitation is the methylation level difference (3.3%) between famine-exposed and non-exposed groups. There is no evidence that such a small methylation difference would affect gene expression and hence phenotypes. Other limitations discussed by the authors include that the DNA methylation analysis was conducted 50 years after fetal exposure to famine, that the exposed and non-exposed groups differed in environmental exposures following the exposure, and that the exposed and non-exposed groups had distinct genetic backgrounds. These limitations need to be considered in future famine or undernutrition studies.

In a different study, the effects of prenatal famine on DNA methylation in the serotonin signaling pathway were examined in 184 individuals born during the famine (1 January 1959, to 31 December 1961) and 105 unexposed controls born after the famine (1 January 1963, to 31 December 1964) (Zeng et al., 2023). This study provided insight into how prenatal famine exposure may affect the epigenetic regulation of genes involved in neurotransmission pathways, potentially influencing long-term health outcomes. Reduced methylation of the 5-hydroxytryptamine receptor 2C gene (HTR2C), which is associated with serotonin signaling, was observed in females (P = 0.014), but no significant methylation differences were detected in males. The authors did not provide the absolute methylation level differences between groups, limiting the ability to assess biological relevance. A major limitation of the study is the reliance on DNA methylation data derived from blood, which may not accurately reflect methylation patterns in brain regions associated with neuropsychiatric disorders. Without validating whether methylation patterns in blood correspond to those in relevant brain tissues, conclusions drawn about the impact of famine exposure on brain-related outcomes remain speculative. Additionally, while the study’s control group consisted of individuals born after the famine, their parents’ germ cells were likely exposed to the same famine conditions. This compromises the distinction between exposed and non-exposed groups, limiting the ability to attribute observed differences solely to *in utero* famine exposure.

Similarly, epigenetic effects of prenatal famine were examined in 25 individuals exposed to the Chinese famine during the first 3 months *in utero* and 54 controls from the same population (He et al., 2019). In addition, the authors analyzed DNA methylation in fibroblasts derived from skin samples of five healthy Dutch individuals, cultured under conditions similar to those of famine. Genome-wide methylation analysis was conducted in blood cells from 79 individuals and in a fibroblast cell sample, followed by pathway enrichment analysis. However, this analysis has not revealed any significant differences in methylation between the two groups. Instead, the authors reported an overlap of three DMRs between blood and cell culture samples. These DMRs were assigned to genes involved in nervous system development and neurogenesis, including ENO2, ZNF226, CCDC51, and TMA7. One key limitation of the study investigating the effects of the Chinese Famine on individuals exposed *in utero* is the use of blood cells for DNA methylation analysis, which the authors acknowledge as suboptimal for capturing relevant epigenetic changes. Additionally, the study suffers from an imbalance in sample sizes, with 54 control individuals compared to only 25 famine-exposed individuals, thereby reducing the analysis’s statistical power. This limitation, combined with overall low power, hindered the ability to detect DMRs with confidence. Furthermore, comparing DMRs from famine-exposed individuals with those from healthy Dutch fibroblasts is methodologically inappropriate, given differences in tissue type and population background. Finally, the absence of phenotypic data from the famine-exposed individuals limits the ability to interpret the functional relevance of any observed epigenetic differences.

The Genomic Research of the Chinese Famine (GRECF) study examined the impact of famine exposure on DNA methylation of the insulin-like growth factor 2 (IGF2) gene and blood lipid levels in adulthood. The study included individuals who experienced famine as fetuses or infants, and those born after the famine (Shen et al., 2019). Blood samples from 180 participants were collected approximately 50 years after the famine and analyzed for DNA methylation. Participants were assigned to moderate or severe famine groups based on their residence in provinces with varying rates of starvation and mortality. Analysis of eight CpG sites revealed only one CpG site associated with severe famine exposure, with a 7% increase in methylation at CpG1 of IGF2 observed in the during-famine cohort. The methylation level of this CpG was associated with elevated cholesterol levels in adulthood. This study has several notable limitations. The observed differences in DNA methylation (7%) were minimal, with only one out of eight examined CpG sites reaching statistical significance. Moreover, the analysis was conducted in blood cells, which the authors acknowledged may not accurately reflect methylation patterns in relevant tissues. Additionally, the study’s limited sample size further reduced its statistical power, compromising the reliability of the findings.

Thus, although epigenetic mechanisms are widely proposed to mediate the intergenerational transmission of phenotypes following parental famine exposure, most studies report associations rather than causal relationships (Boks et al., 2018; He et al., 2019; Li et al., 2021; Shen et al., 2019; Tobi et al., 2009; Wang et al., 2019; 2020; Zeng et al., 2023). In contrast, Allard et al. (2015) provided evidence supporting a causal link between maternal glycemia and epigenetic regulation of leptin in offspring. Higher maternal fasting glucose was associated with increased neonatal cord blood leptin levels and with altered DNA methylation at the leptin gene locus, potentially contributing to long-term programming of excess adiposity. Furthermore, the only epigenetic modification examined in these studies was DNA methylation. Therefore, there is a need to investigate additional epigenetic mechanisms, including histone modifications, non-coding RNAs, and chromatin structure.

Other epigenetic studies have reported methylation differences using percentages or P values, which may be statistically significant but fail to convey the biological relevance of the findings (Tobi et al., 2014). Thus, as many epigenetic epidemiology studies report only modest differences in DNA methylation between cases and controls, the key challenge is determining whether these differences are biologically meaningful. Accumulating evidence suggests that even small methylation changes can have functional significance, particularly when they occur in regulatory regions of the genome or are consistently replicated across independent studies and populations. One reason small methylation differences may be relevant is their localization within regulatory elements, such as promoters, enhancers, and transcription factor binding sites. For example, DNA methylation (5 mC) at enhancers is highly dynamic across cell types, decreasing when enhancers are active and increasing upon inactivation (Kreibich and Krebs, 2023). Active enhancers also undergo continuous TET-mediated demethylation, leading to intermediate methylation levels. These patterns indicate that 5 mC plays a key role in regulating enhancer and promoter activity and, consequently, cell type–specific gene expression programs (Kreibich and Krebs, 2023). Another line of evidence supporting the biological relevance of small methylation changes comes from studies demonstrating the replication of epigenetic signals across independent datasets (Joubert et al., 2016). These observations indicate that small differences in DNA methylation should not be dismissed as biologically trivial. However, current epidemiological famine studies lack in-depth analyses of the genomic localization of DNA methylation changes within regulatory elements.

In summary, across famine studies, several associations have been reported between DNA methylation changes and later-life health outcomes, particularly in metabolic pathways, although the evidence remains largely correlational and modest in magnitude. For example, prenatal exposure to the Chinese famine has been linked to increased DNA methylation of the INSR gene, which was associated with greater waist circumference, higher triglyceride levels, and lower HDL cholesterol in adulthood, suggesting a potential role in metabolic dysfunction (Wang et al., 2019). Similarly, altered methylation of IGF2 has been associated with elevated cholesterol levels, further implicating epigenetic variation in lipid metabolism (Shen et al., 2019). Overall, while these findings suggest that famine-associated DNA methylation changes may be linked to metabolic and, potentially, neurodevelopmental outcomes, the observed effects are generally small, tissue-specific, and based on associations rather than causal evidence, highlighting the need for more robust and functionally validated studies.

To enhance interpretability, future studies should incorporate measures that combine statistical significance with biologically meaningful metrics. For example, reporting absolute and relative methylation differences, effect sizes, or fold changes would provide deeper insights into the functional implications of prenatal famine exposure.

### Famine intensity and later-life phenotypes

Several famine studies have moved beyond simple binary exposure classifications to examine famine intensity, providing important evidence for dose–response relationships and underscoring the value of incorporating severity measures in future research. For example, Wang et al. (2017) assessed the effects of prenatal and postnatal exposure to varying degrees of the Chinese famine and reported that severe exposure during fetal life and childhood was associated with higher HbA1c levels and an increased prevalence of diabetes in adulthood. However, the absence of an unexposed control group limits the strength of causal inference. Similarly, Fransen et al. (2016) identified dose-dependent associations between early-life famine exposure and adult lifestyle factors, with more severe exposure linked to higher prevalence of smoking and physical inactivity, although no consistent associations were observed for alcohol consumption or diet. This study was limited by potential recall bias, particularly among younger women who may have relied on family reports of famine exposure.

More recently, Lumey et al. (2024) demonstrated a clear dose–response relationship between famine severity and later-life health outcomes in the Holodomor, where intensity was defined using excess mortality. Their findings showed that more severe exposure during early gestation was associated with a progressively higher risk of type 2 diabetes in adulthood. Collectively, these studies highlight that incorporating well-defined measures of famine severity, whether based on regional indicators or individual experiences, can substantially improve causal inference and provide deeper insight into the long-term biological and health consequences of early-life undernutrition.

### Lessons learned and current famines in Sudan, Somalia, and Palestine

The Dutch and Chinese famines differed markedly in duration, geographic variability, and definition, which has important methodological implications. The Dutch Hunger Winter (1944–1945) was short, well-documented, and geographically confined, allowing precise timing of exposure. In contrast, the Chinese famine (1959–1961) was prolonged, heterogeneous across provinces, and varied substantially in severity between rural and urban regions. These differences complicate exposure classification, control selection, and causal inference, underscoring distinct methodological challenges in studying each famine.

The Dutch and Chinese famines have offered valuable insights into the long-term health consequences of *in utero* nutritional deprivation. These studies support the hypothesis that early-life famine exposure can result in lasting epigenetic modifications, contributing to an increased risk of metabolic, cardiovascular, and neuropsychiatric disorders. However, this body of research faces several important limitations, including technical challenges in data collection, limited accuracy of historical information, and suboptimal experimental designs. Many studies were conducted decades after the exposure period and, in some cases, lacked precise or well-defined phenotypic data. Inadequate selection of control groups, particularly when individuals were born shortly after the famine ended, raises concerns about residual maternal stress exposure. Moreover, variation in tissue types, small sample sizes, and inconsistent phenotypic measures further reduce statistical power and hinder meaningful comparisons across studies.

Current and recent famines in Sudan, Somalia, and Gaza in Palestine, caused by wars and environmental factors such as droughts and floods, are among the most serious humanitarian crises in recent history. In 2011–12, Somalia experienced the most severe famine of the twenty-first century, resulting in an estimated 258,000 deaths. This devastating famine was caused by a combination of extreme drought, major agricultural failures, a global surge in food prices that sharply diminished purchasing power, and ongoing conflict (Maxwell et al., 2016). At present, approximately 18 million people in Sudan are suffering from acute hunger, with five million facing emergency levels (www.wfp.org). Furthermore, Sudan is grappling with the worst displacement crisis globally, where over nine million people have been displaced due to conflict, and more than two million have sought refuge in neighboring countries (www.wfp.org).

Multiple United Nations (U.N.) agencies have reported that since October 2023, the Palestinian population in Gaza has been facing hunger, stress, trauma, and a scarcity of healthcare services. According to the U.N. World Food Program (WFP) USA, the displacement of over 90% of Gaza’s population, the destruction of food production and farmland, and restrictions on food imports have resulted in profound food insecurity across the entire population of 2.2 million people, elevating the risk of famine (www.wfpusa.org). According to the Integrated Food Security Phase Classification (IPC) report, approximately 50% of Gaza’s population is currently in the emergency phase, facing acute malnutrition, while around 25% is experiencing extreme food scarcity (www.wfpusa.org). The IPC serves as the principal framework used by the international community to assess the severity and scale of hunger crises against established scientific criteria. The widespread food insecurity in Gaza’s entire population poses differential risks to individuals depending on developmental stage, with fetuses and their germ cells bearing the potential for lifelong detriment deriving from a nutrient-deficient *in utero* environment. Other critical periods of early development are also highly vulnerable to environmental stressors that may influence disease susceptibility, such as pre-puberty (Skinner, 2011). Consequently, hunger and other factors may have a more profound impact on children in Gaza, with additional potential for detrimental effects on their future offspring.

Alongside shortages in food, clean water, medicines, and access to adequate healthcare, children in Gaza have endured significant traumatic events, such as repeated displacements, bombings, and parental separation. The United Nations Children’s Fund (UNICEF) approximates that at least 17,000 children have been separated from their parents, with nearly all children in Gaza requiring mental health and psychological support (www.unicefusa.org). These children exhibit symptoms including increased anxiety levels, loss of appetite, sleep disturbances, and panic attacks (www.unicefusa.org). Yehuda and colleagues explored intergenerational transmission of epigenetic markers in response to Holocaust trauma (Yehuda et al., 2016). Exposure to the Holocaust—whether through detention in a concentration camp or witnessing or experiencing torture—was linked to elevated methylation levels in the FK506 binding protein 5 (*FKBP5*) gene (Yehuda et al., 2016). Additionally, alterations in DNA methylation within *FKBP5* were noted in the children of Holocaust survivors (Yehuda et al., 2016). While this study has certain limitations, including a small sample size, the use of blood cells for methylation analysis, and a limited number of genes, its findings reveal notable epigenetic effects in the offspring of Holocaust survivors. Indeed, to thoroughly investigate transgenerational epigenetic inheritance, it is essential to determine whether sperm or egg cells carry the epigenetic marks altered in response to environmental stimuli. The question of whether offspring can inherit effects of parental experiences was explored in mice (Dias and Ressler, 2014). The researchers exposed F0 mice to odor fear conditioning before mating and observed that subsequently conceived F1 and F2 generations displayed heightened behavioral sensitivity to the F0-conditioned odor. Notably, the inheritance of this behavior was concomitant with the transmission of DNA methylation alterations in the Olfr151 odorant receptor gene, which were detected in the sperm of both the conditioned F0 and F1 generations (Dias and Ressler, 2014).

Human and animal studies offer substantial evidence supporting the concept that parental experiences, including trauma and behavior, can be transmitted to subsequent generations, potentially mediated by epigenetic mechanisms. Therefore, it is imperative to investigate the phenotypic and epigenetic impacts of undernutrition, trauma, and stress in the vulnerable population of Gaza, Sudan, and Somalia, particularly among children and pregnant women. Unlike investigations of the epigenetic effects of the Dutch and Chinese famines, which occurred several decades later, earlier initiation of such studies could yield more reliable data with fewer confounding factors. Conducting studies earlier may offer several advantages, including more precise definitions of famine or trauma exposure, access to a broader range of tissue samples, larger sample sizes, and better-defined control groups. Additionally, earlier studies could help identify potential interventions for affected individuals later in life, as well as for their offspring. Epigenetic research has the potential to identify both resilience-promoting factors and vulnerability factors in response to trauma and famine. Understanding these factors can inform interventions aimed at enhancing resilience and alleviating the adverse effects of trauma on children’s health and development. Furthermore, studying epigenetic effects can offer insights into the biological mechanisms through which trauma and famine impact individuals, leading to more targeted and effective interventions for children who have endured the stress of war. Ultimately, such research can shed light on how trauma and undernutrition experienced during conflict can have enduring effects on health and wellbeing.

### Future directions

Although studies of famine exposure have yielded key insights into developmental programming and long-term disease risk, genome-scale epigenetic analyses remain limited. Early human research largely relied on targeted loci, yet provided compelling evidence for persistent, environmentally induced epigenetic variation. Notably, studies of individuals prenatally exposed to the Dutch Hunger Winter identified lasting DNA methylation differences at the imprinted *IGF2* locus decades later, demonstrating that early-life nutritional deprivation can leave stable epigenetic marks (Heijmans et al., 2008).

A deeper mechanistic understanding will require broader, integrative approaches. Advances such as epigenome-wide association studies (EWAS), high-throughput methylation profiling, and multi-omics integration now enable unbiased, genome-scale discovery of environmentally responsive epigenetic variation. EWAS interrogates hundreds of thousands of CpG sites, while integration with transcriptomic, proteomic, and metabolomic data helps link epigenetic changes to molecular pathways and disease phenotypes. These strategies are central to modern epigenetic epidemiology, which emphasizes large datasets, replication, and integrative analyses to dissect interactions between environmental exposures and gene regulation (Rakyan et al., 2011).

However, applying such approaches to famine cohorts remains challenging. Historical exposures often involve small sample sizes, heterogeneous contexts, and limited availability of relevant biological samples. Multi-omics integration further requires substantial resources, harmonized study designs, and advanced statistical methods to address cellular heterogeneity, confounding, and modest effect sizes. Despite these challenges, incorporating EWAS, multi-omics, and high-throughput methylation technologies will be critical for improving the resolution, reproducibility, and mechanistic insight of famine-associated epigenetic signatures.

Despite increasing evidence linking early-life famine exposure to persistent epigenetic variation, the robustness of these findings is limited by modest sample sizes, cohort-specific designs, and restricted replication. Many famine studies, for example, those examining DNA methylation changes associated with prenatal exposure in cohorts from the Dutch Hunger Winter or the Chinese Great Famine, have relied on relatively small numbers of individuals and targeted loci, limiting statistical power and increasing susceptibility to cohort-specific confounding. Epigenetic epidemiology faces challenges similar to those encountered in early genetic association studies, including small effect sizes, cellular heterogeneity, and environmental confounders, highlighting the need for large sample sizes and rigorous replication across independent populations. Consequently, large collaborative consortia, harmonized cohort designs, and meta-analyses across diverse populations will be essential to establish reproducible epigenetic signatures of famine exposure and to distinguish causal mechanisms from cohort-specific or stochastic variation. Such approaches have already proven transformative in genomics and are likely to be equally important for improving the reliability and interpretability of future human famine studies.

## Conclusion

In conclusion, future post-famine research should prioritize more rigorous experimental designs to enhance reliability and minimize bias. This includes selecting truly unexposed control groups born before the onset of famine, precisely defining exposure timing, using tissues relevant to the studied phenotype, and incorporating both molecular and physiological outcome measures. Studies should also report relevant methylation changes and validate their biological impact through gene expression or functional assays. Larger, multi-cohort analyses and longitudinal designs would enhance statistical power and allow for examination of transgenerational effects. Furthermore, future famine studies could benefit from new genomic technologies and advanced methods, such as genome-wide analyses, multi-omics approaches, and machine learning, to gain deeper insights into the biological consequences of early-life exposure to famine. By establishing these standards, future work can better clarify the role of early nutritional deprivation in shaping lifelong health and contribute more directly to evidence-based public health interventions. More accurate research outcomes can help inform public policy and interventions, such as nutritional supplementation for pregnant women in food-insecure or famine-affected regions.
